# The promoting effect and mechanism of Nrf2 on cell metastasis in cervical cancer

**DOI:** 10.1186/s12967-023-04287-0

**Published:** 2023-07-04

**Authors:** Mengwen Zhang, Xiaoling Hong, Ning Ma, Zhentong Wei, Xinxin Ci, Songling Zhang

**Affiliations:** 1 Department of Obstetrics and Gynecology, The First Hospital of Jilin University, Changchun, China; 2 Institute of Translational Medicine, The First Hospital of Jilin University, Changchun, China

**Keywords:** Nuclear factor erythroid 2-related factor 2 (Nrf2, Nfe2l2), Epithelial-mesenchymal transition (EMT), Anoikis, Neoplasm metastasis, Cervical cancer (CC)

## Abstract

**Background:**

Cervical cancer (CC) has poor prognosis and high mortality rate for its metastasis during the disease progression. Epithelial-mesenchymal transition (EMT) and anoikis are initial and pivotal steps during the metastatic process. Although higher levels of Nrf2 are associated with aggressive tumor behaviors in cervical cancer, the detailed mechanism of Nrf2 in cervical cancer metastasis, especially EMT and anoikis, remains unclear.

**Methods:**

Immunohistochemistry (IHC) was used to examine Nrf2 expression in CC. Wound healing assay and transwell analysis were used to evaluate the migration ability of CC cells. Western blot, qTR-PCR and immunofluorescent staining were used to verify the expression level of Nrf2, the EMT associated markers and anoikis associated proteins. Flow cytometry assays and cell counting were used to detect the apoptosis of cervical cancer cells. The lung and lymph node metastatic mouse model were established for studies in vivo. The interaction between Nrf2 and Snail1 was confirmed by rescue-of-function assay.

**Results:**

When compared with cervical cancer patients without lymph node metastasis, Nrf2 was highly expressed in patients with lymph node metastasis. And Nrf2 was proved to enhance the migration ability of HeLa and SiHa cells. In addition, Nrf2 was positively correlated with EMT processes and negatively associated with anoikis in cervical cancer. In vivo, a xenograft assay also showed that Nrf2 facilitated both pulmonary and lymphatic distant metastasis of cervical cancer. Rescue-of-function assay further revealed the mechanism that Nrf2 impacted the metastasis of CC through Snail1.

**Conclusion:**

Our fundings established Nrf2 plays a crucial role in the metastasis of cervical cancer by enhancing EMT and resistance to anoikis by promoting the expression of Snail1, with potential value as a therapeutic candidate.

## Introduction

Cervical cancer (CC) is the fourth most frequently diagnosed malignant disease among women worldwide, and the mortality rate is higher in low- and middle-income countries [1]. The poor prognosis and high mortality rate of CC can be attributed to its metastasis, which mainly involves direct spread and lymph node metastasis. Previous studies have shown that the basic events of malignant cell metastasis include the following behaviors: losing adhesion among cells and the surrounding matrix, gaining the characteristics and ability to invade surrounding tissues, resisting anoikis in blood or lymphatic vessels, and proliferation in a suitable place [2–4]. However, the detailed mechanisms of metastasis in cervical cancer are highly complex biological processes [5] and have not been fully elucidated. Therefore, potential therapeutic targets in metastasis need to be explored specifically.

Although originally developed in the field of embryology, many studies have verified that epithelial-mesenchymal transition (EMT) plays a key role in cancer metastasis [6]. Some studies have reported that regulating EMT-associated molecules such as E-cadherin (E-cad), Snail (Snail1), Slug (Snail2) and Vimentin could promote the invasion and metastasis of cervical cancer cells in vitro and in vivo.[7–9]. In addition, anoikis ensures the elimination of misaligned cells and prevents distant metastasis of malignant cells. Previous studies have verified that the expression of focal adhesion kinase (FAK) plays a protective role in resisting anoikis in malignancy [10, 11]. In cervical cancer, enhancing anoikis resistance significantly enhanced migration and invasion in vivo and in vitro [12]. However, it remains unknown how EMT and anoikis result in the metastatic progression of cervical cancer.

Nuclear factor erythroid 2-related factor 2 (Nrf2) is a transcription factor and regulator of many antioxidant enzymes and cell-protective genes [13]. Previous studies suggested that cancer can be driven and even promoted by chromosomal abnormalities and oncogene activation determined by oxidative stress [14, 15]. Mechanically, oxidative stress causes damage to DNA, proteins, lipids, and the initiation of cell death ultimately. To maintain redox homeostasis and prevent the harmful consequences of oxidative stress, cancer cells upregulate a network of reactive oxygen species (ROS) scavenging enzymes and antioxidant pathway [16]. Nrf2 plays an important controller of the enzymes and antioxidant genes in the network. And, the activation of Nrf2 is usually described as uniquely dualistic and can resist cellular malignancy before cancer formation; however, its high expression during cancer progression will promote the development of carcinoma [17, 18]. In addition, chemotherapy and radiotherapy produce high levels of oxidative stress in tumors accompanied by DNA damage, which is a possible reason for the frequent occurrence of resistance in several cancers, such as ovarian cancer [19], breast cancer [20], cervical and endometrial cancers [21] and so on. Some studies have shown that the activation of the Nrf2 signaling pathway could promote the EMT process in hepatocellular carcinoma cells and lung cancer cells [22, 23]. In addition, the increased level of heme oxygenase 1 (HO-1), which was caused by activation of Nrf2, could prevent anoikis and promote metastasis in carcinoma upon suspension [24]. Ma et al. showed that the level of Nrf2 was higher in cervical cancer compared with normal or pro-cancer patients. They considered that the increased Nrf2 endowed the proliferation, migration and invasion of cervical cancer cells [25]. Yet, the molecular mechanisms through which Nrf2 regulates EMT and anoikis in the metastasis of cervical cancer are still not clear.

In the present study, we first confirmed that Nrf2 expression levels were higher in cervical cancer patients with lymph node metastasis. We subsequently established Nrf2 knockout cell lines and Nrf2 overexpression cells to explore Nrf2’s role in CC cells and in mice. Our results showed that Nrf2 overexpression could significantly promote the transformation of the epithelial phenotype to the mesenchymal phenotype in cervical cancer cells. Moreover, the Nrf2 signaling pathway was activated in suspension, and the related proteins of apoptosis and anoikis changed observably. Mechanistically, the results of rescue-of-function assay showed that Nrf2 could facilitate the migration of cervical cancer cells by accelerating EMT and resisting anoikis through promoting the expression of Snail1. Consequently, it suggested that Nrf2 could be used as a potential therapeutic target for CC with metastasis.

## Materials and methods

### Clinical specimens

The clinical specimens were obtained from ten pairs of cervical cancer patients with or without lymph node metastasis confirmed by postoperative pathological results from 76 cervical cancer patients at the First Hospital of Jilin University from January 2019 to December 2021. All these specimens were collected after approval by the Ethical Committee of the First Hospital of Jilin University. Informed consent was obtained from patients’ surrogates. None of these patients had received preoperative radiation or chemotherapy before the specimens were collected.

### Immunohistochemistry (IHC)

The tissue specimens were fixed in 10% neutral formalin (room temperature, 20 min), embedded in paraffin, and prepared in 4-mm-thick sections. Next, paraffin wax-embedded tissue sections were dewaxed, rehydrated, microwaved and blocked endogenous peroxidase activity. Finally, the tissue sections were incubated with the primary antibody against Nrf2 (product code: ab137550, 1:500 dilution, Abcam Biotech Corp., CSP, UK) at 4 ℃ overnight. The staining score was reviewed by two different pathologists. Adding the percentage of positive cells (scored as 1, 0–25%; 2, 26–50%; 3, 51–75%; 4, > 75%) and the staining intensity (scored as 0, negative; 1, light yellow, weakly positive; 2, medium yellow, moderately positive; 3, brown, strongly positive).

### Cell lines and cell culture

Human cervical cancer HeLa and SiHa cells were purchased from the American Type Culture Collection (ATCC, Manassas, VA, USA) and cultured in Eagle’s Minimum Essential Medium (EMEM, Catalog No. 30-2003) supplemented with 1% penicillin/streptomycin (Service Bio., Wuhan, China) and 10% fetal bovine serum (FBS, B.I. Co., USA), 37 ℃, 5% CO_2_.

### Nrf2 knockout in HeLa cell

The internet tool (http://crispr.mit.edu) from Feng Zhang Lab at Harvard University was used to design the sgRNA of Nrf2 and send the primers to Comate Biotechnology Company (Changchun, Chain) for synthesis. The sequences of the primers are shown in Table 1. Upstream and downstream chains (10 μmol/l) were connected to the linearized carrier obtained from Transgen Company (Beijing, China). The ligand product, 1 ml, was used to transform the Trans 5α chemically competent cells and screened with ampicillin resistance plates and sequenced at Comate Biotechnology Company. HeLa cells were transfected and then selected by puromycin 2 μg/ml purchased from Sigma-Aldrich (USA). Photographs of Nrf2 knockout cells were taken with microscopic imaging system under an inverted microscope (Olympus, China).

**Table 1:** Sequence Information

Name	Sequence (5′ → 3′)
Nrf2-sgRNA1F	CACCGCGACGGAAAGAGTATGAGC
Nrf2-sgRNA1R	AAACGCTCATACTCTTTCCGTCGC
Nrf2-sgRNA2F	CACCGTCGATGTGACCGGGAATATC
Nrf2-sgRNA2R	AAACGATATTCCCGGTCACATCGAC

### Transient overexpression of Nrf2

Nrf2-specific or VR1012 plasmids were purchased from Comate Biotechnology Company (Jilin, China), cotransfected with PEI into HeLa and SiHa cells and cultured with fresh serum-free EMEM at 37 ℃ in an incubator containing 5% CO_2_. After 2 h, the same volume of EMEM supplemented with 20% FBS was added.

### qRT-PCR

Total RNA was isolated from cell lines using TRIzol reagent (Invitrogen, USA). RNA concentration was determined using a Synergy^™^ H1 spectrophotometer (BioTek Instruments, Inc., USA). Reverse transcription was performed using Prime Script RT Master Mix (Transgen Biotech, Beijing, China). qRT-PCR was performed with SYBR Premix Ex Taq (TaKaRa Bio., Beijing, China) using Applied Biosystems Quant Studio 5 (Thermo Fisher Scientific, Shanghai, China). Fold changes in Nrf2 expression were calculated using the 2^−ΔΔCt^ method and normalized to β-actin expression. The primers are presented as Table 2.

**Table 2:** Sequence Information

Name	Sequence (5′ → 3′)
human Nrf2-F	CCAATTCAGCCAGCCAGCACAT
human Nrf2-R	CAGGTGACTGAGCCTGATTAGTAG
human β-actin-F	CATGTACGTTGCTATCCAGGC
human β-actin-R	CTCCTTAATGTCACGCACGAT

### Western blot analysis

Appropriate cells were collected, and cell lysates (Beyotime Biotechnology, China) were used to extract proteins. Samples were added to gels for electrophoresis, transferred to polyvinylidene fluoride membranes using a semidry apparatus (Bio-Rad, USA), blocked with 5% nonfat milk for 1 hour at room temperature and incubated overnight with appropriately diluted primary antibodies at 4 ℃. Antibodies against GCLC (product code: ab207777, 1:1000 dilution), HO-1 (ab52947, 1:1000 dilution), GCLM (ab126704, 1:10,000 dilution) and NQO1 (ab80588, 1:20,000 dilution) were purchased from Abcam Biotech Corp. (CSP, UK). Antibodies against E-cad (Cat No. 20874-1-AP, 1:20,000 dilution), N-cad (22,018-1-AP, 1:2000 dilution), Vimentin (10,366-1-AP, 1:2000 dilution), Snail1 (13,099-1-AP, 1:500 dilution), PARP1 (13,371-1-AP, 1:1000 dilution) and β-actin (20,536-1-AP, 1:3000 dilution) were obtained from Proteintech Group, Inc. (Chicago, IL, USA). Antibodies against Slug (product code: 9585 T, 1:1000 dilution), Caspase-3 (9668 T, 1:1000 dilution), Bax (5023 T, 1:1000 dilution), FAK (71433 T, 1:1000 dilution), Bcl2 (4223 T, 1:1000 dilution) and Smad (8685 T, 1:1000 dilution) were purchased from Cell Signaling Technology, Inc. (MA, USA). The membranes were washed 3 times by PBST and then incubated in alkaline phosphatase-conjugated secondary antibodies (33101ES60 and 33202ES60, 1:5000 dilution, Yeasen Biotechnology (Shanghai) Co., Ltd.) at room temperature for 1 h. Above membranes were washed again and staining was conducted by the BCIP/NBT Alkaline Phosphatase Color Development kit (Zoman Bio., Co., Beijing, China).

### Wound healing assay

Cells were cultured in 12-well plates to complete confluence, and straight wounds were generated by 200-μl pipette tips. Using PBS to remove exfoliated cells and adding fresh serum-free EMEM. The wounds were photographed at 0 and 48 h, and the gaps were analyzed by ImageJ software. The formula for calculating mobility was as follows: ratio of migration = [(gap width at 0 h)—(gap width at 48 h)]/(gap width at 0 h) × 100.

### Transwell analysis

Twenty-four-well plates and Transwells were purchased from Corning-Costar, USA. Cells were seeded and cultured with fresh serum-free EMEM in upper Transwells house, and the lower 24-well plates containing EMEM supplemented with 20% FBS. After 48 h, the upper houses were washed with PBS and fixed with 4% methanol. The nonmigrated cells were removed by PBS, and the migrated cells were stained with crystal violet. Five representative pictures were chosen, and the cell numbers were counted.

### Immunofluorescent staining

For immunofluorescence staining, cells were cultured on autoclaved coverslips, fixed with 4% paraformaldehyde, permeabilized with 0.5% Triton X-100, and incubated with primary antibodies (E-cad: 20,874-1-AP, 1:500 dilution; Vimentin: 10,366-1-AP, 1:500 dilution; Proteintech Group, Inc., Chicago, IL, USA) at 4 ℃ overnight. On the next day, the coverslips were washed 3 times using TBST, incubated with goat anti-mouse Alexa Fluor^®^ 594 or goat anti-rabbit Alexa Fluor^®^ 488 (ZSGB Bio., Beijing, China) for 1 h at room temperature in dark, washed 3 times again and photographed.

### Anoikis model and cell counting

Poly-2-hydroxyethyl methacrylate (12 mg/ml) was obtained from Sigma‒Aldrich (USA) in 95% ethanol under ultraviolet light in an ultraclean platform at room temperature for drying. Cells (2 × 10^4^) were placed in suspension culture on cell culture plates as described above to establish the model of anoikis [26, 27]. Cell counting was performed for 7 consecutive days, and cell survival rates were generated via GraphPad Prism software.

### Flow cytometry assays

An Annexin V-APC/7-AAD apoptosis kit (Multi Sciences Biotech Co., Hangzhou, China) was used to detect the percentage of cell apoptosis according to the manufacturer’s instructions. Cells were suspended at a concentration of 1 × 10^5^ cells/ml with cold binding buffer. 5 μl of Annexin V-APC and 10 μl of 7-AAD were added to the cell suspension, blended gently and incubated in the dark. The samples were analyzed by a Cantoll flow cytometer (BD Biosciences, FACS, USA) within 1 h.

### Si-RNA

Si-RNA targeting Snail1 and the transfection reagent RNA Fit were obtained from HANBIO, Shanghai, China. Transfection of si-RNA was performed when cells reached 60% confluency. The final concentration of si-RNA was 20 nmol/ml, and detected silencing efficiency through western blot after 72 h.

### In vivo metastasis assays and hematoxylin–eosin staining

BALB/c-nu mice (female, 4–5 weeks old, weighing 13–16 g) were purchased from Vital River Animal Co., Ltd. (Beijing, China). All animal studies were approved by the Medical Experimental Animal Care and Use Committee of Jilin University. For lung metastasis mouse models, the mice were randomly separated into two groups (n = 6/group) and injected with 1.5 × 10^6^ cells/150 µl (HeLa-sg-Ctrl; HeLa-sg-Nrf2) through tail vein. After 45 days, the mice were sacrificed, and their lungs were excised to hematoxylin–eosin (H&E) staining [28]. For the lymph node metastatic mouse model, the mice were injected with 1 × 10^7^ cells/50 µl into the foot pads and were euthanized on Day 45. Macroscopic lymph nodes were counted, collected and subjected to H&E staining to determine the lymph node metastasis rate.

### Data analysis

All studies were repeated at least 3 times. The data were analyzed by SPSS 26.0 (IBM) and presented as the mean ± standard error of means. The experimental data between two groups were compared by Student’s t tests. *P* < 0.05 was considered statistically significant.

## Results

### Nrf2 was more highly expressed in patients with positive lymph node metastasis.

**Fig. 1:**
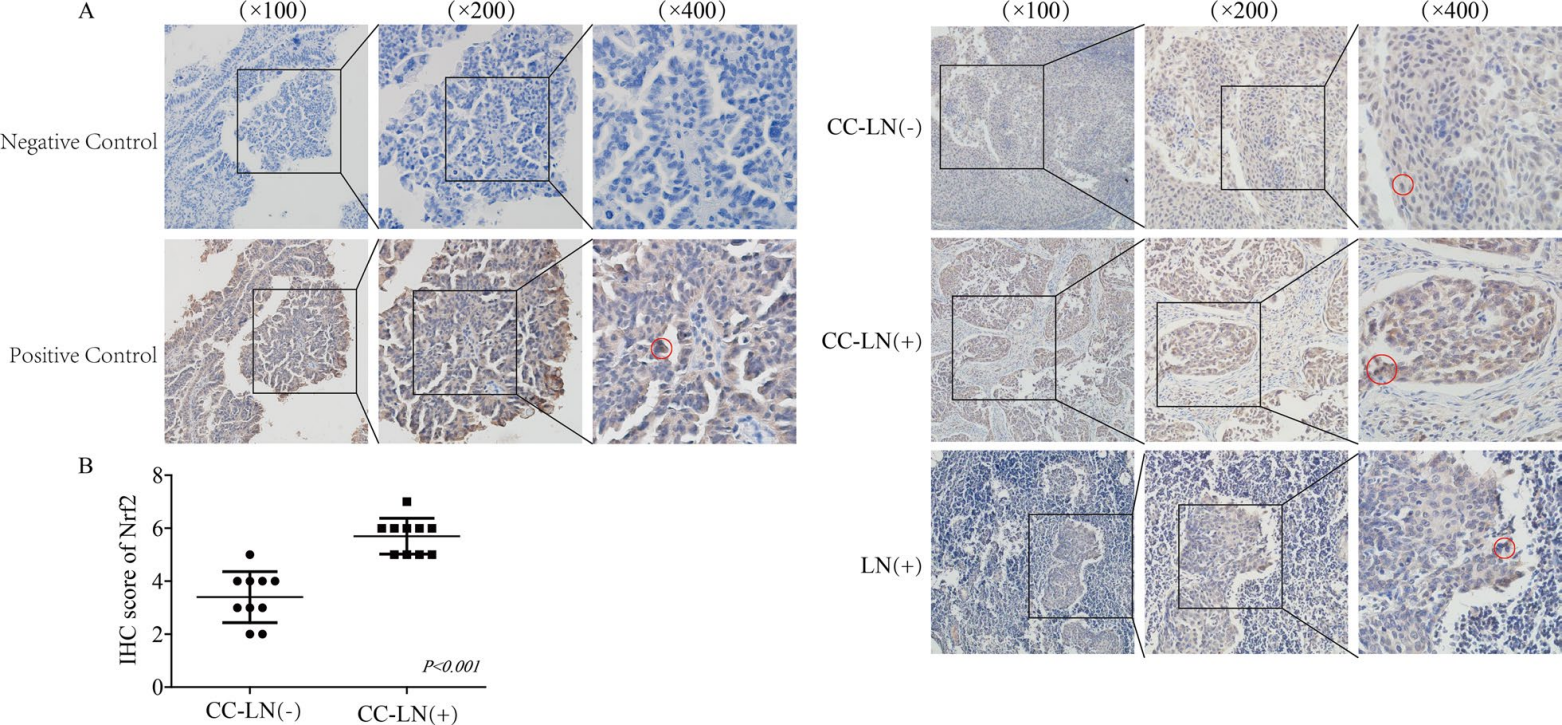
The expression level of Nrf2 was increased significantly in cervical cancer with lymph node metastasis. **A** Representative IHC images of Nrf2 in cervical cancer with or without lymph node metastasis and lymph nodes in patients. The red circle shows the typical IHC entity. **B** The protein level of Nrf2 was significantly increased in patients with lymph node-positive metastasis (n = 10)

Previous studies have shown that the nuclear expression level of Nrf2 was significantly higher than that of normal cervical epithelium [29]. In our study, compared with that in lymph node-negative cervical cancer patients, the level of Nrf2 was increased in lymph node-positive cervical cancer patients (Fig. 1). Therefore, we hypothesized that Nrf2 plays an important role in the process of metastasis in cervical cancer.

### The expression level of Nrf2 correlated with cell metastasis in cervical cancer.

**Fig. 2:**
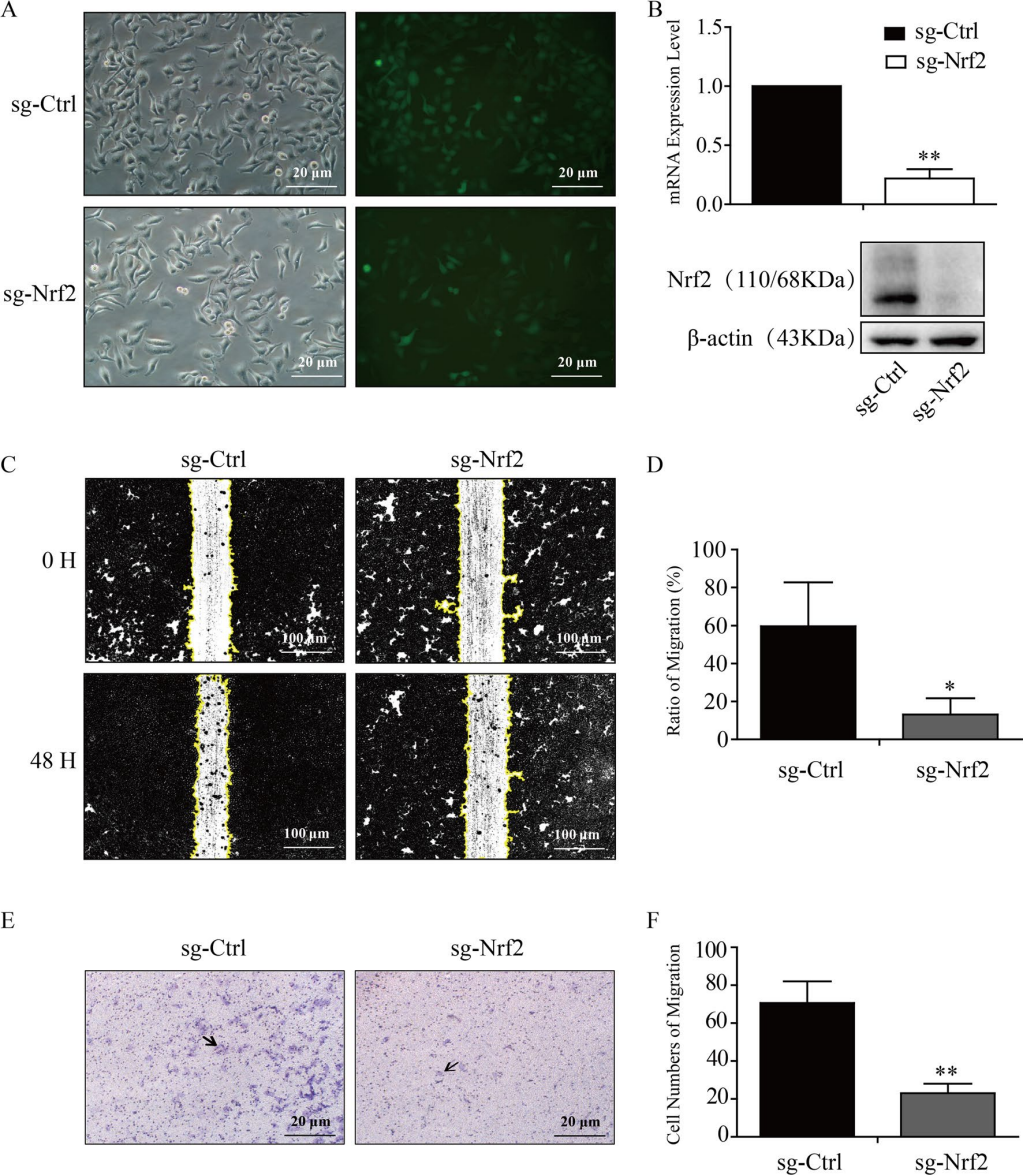
Nrf2 knockout inhibited the migration of HeLa cells.** A** Representative microscopic and fluorescent image of HeLa Sg-Ctrl and HeLa sg-Nrf2 cells are shown. **B** The result of Nrf2 knockout in HeLa cells through western blot analysis and qRT-PCR. **C**, **D** Wound healing assays. **E**, **F** Transwell cell migration experiments. * P < 0.05, ** P < 0.01 (n = 3)

**Fig. 3:**
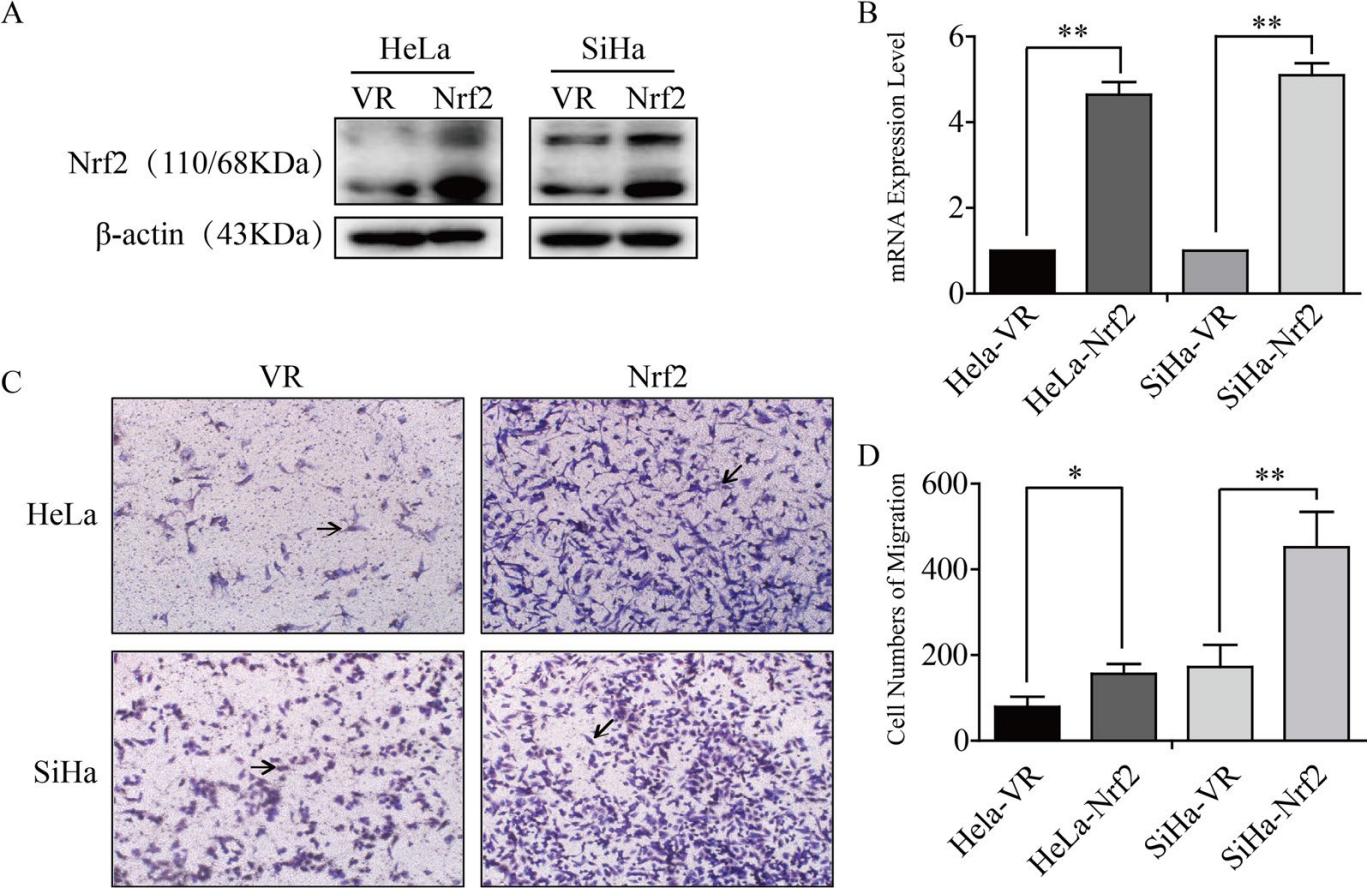
Nrf2 overexpression accelerated the migration of HeLa and SiHa cells. **A** Nrf2 was overexpressed in HeLa and SiHa cells, and the effect was verified by western blotting. **B** The mRNA level of Nrf2. **C**, **D** Transwell cell migration experiments. * P < 0.05, ** P < 0.01 (n = 3)

Here, we constructed a stable Nrf2 knockout cell line in cervical cancer HeLa cells (sg-Ctrl and sg-Nrf2) and verified the knockout efficiency by western blot analysis and qRT-PCR (Fig. 2A, B). Next, we confirmed the effect of Nrf2 on cell metastasis in cervical cancer cells through wound healing assays. Our results showed that the cell mobility of HeLa sg-Ctrl and HeLa sg-Nrf2 cells was 59.7 ± 13.3 (%) and 13.2 ± 3.9 (%), respectively (Fig. 2C, D). Transwell cell migration experiment also showed a similar result: the cell migration numbers of HeLa sg-Ctrl and HeLa sg-Nrf2 were 70.7 ± 6.6 and 23.0 ± 2.9, respectively (Fig. 2E, F). These findings indicated that Nrf2 knockout inhibited the cell metastasis ability in HeLa cervical cancer cells. However, compared with the VR group, the number of migrating cells was increased significantly in Nrf2-overexpressing HeLa and SiHa cells, which were 79.0 ± 13.8 and 172.0 ± 29.9 in HeLa cells and 156.3 ± 13.3 and 451.7 ± 47.8 in SiHa cells, respectively (Fig. 3). Collectively, the above results demonstrated that Nrf2 was positively correlated with cell metastasis ability in HeLa and SiHa cells.

### Nrf2 affected the EMT process in cervical cancer cells.

**Fig. 4:**
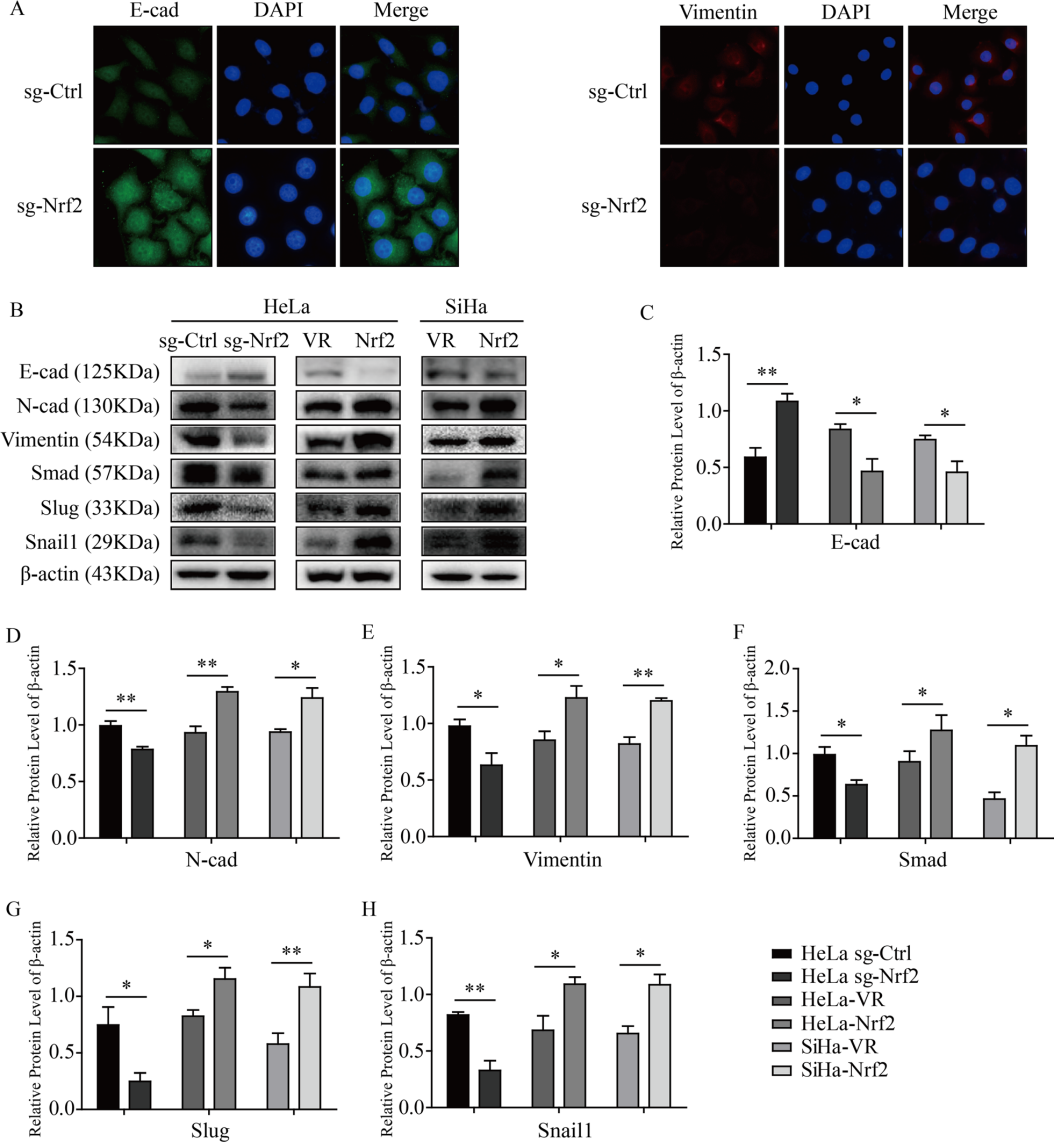
Nrf2 influenced the process of EMT in HeLa and SiHa cells. **A** E-cad and Vimentin were detected by immunofluorescent staining in HeLa sg-Ctrl and HeLa sg-Nrf2 cells. **B** Levels of EMT-associated proteins, including E-cad, N-cad, Snail1, Slug, Smad and Vimentin, were confirmed by western blot analysis. **C-H** The protein expression levels were quantitatively analyzed. * P < 0.05, ** P < 0.01 (n = 3)

In cancer cells, the premise condition of distant metastasis is transforming the original cellular morphological characteristics to gain the corresponding athletic ability; this process is called epithelial-mesenchymal transition. To explore the relationship between Nrf2 and the EMT process in CC, we first measured the expression levels of E-cad and Vimentin, which are representative molecules of the epithelium and mesenchyme, respectively. As shown by immunofluorescence staining, the fluorescence intensity of E-cad increased significantly in sg-Nrf2 HeLa cells compared with sg-Ctrl cells. However, the fluorescence intensity of Vimentin decreased obviously in sg-Nrf2 HeLa cells (Fig. 4A). Next, we measured the levels of EMT-associated proteins, and we found that the level of E-cad was increased significantly, but N-cad, Snail1, Slug, Smad and Vimentin was decreased significantly in the situation of Nrf2 knockout. However, when Nrf2 was overexpressed, the expression levels of above proteins were markedly different in HeLa and SiHa cells (Fig. 4B–H). These results suggested that Nrf2 played an important role in promoting EMT in HeLa and SiHa cells.

### Constructing the model of anoikis in HeLa and SiHa cervical cancer cells.

**Fig. 5:**
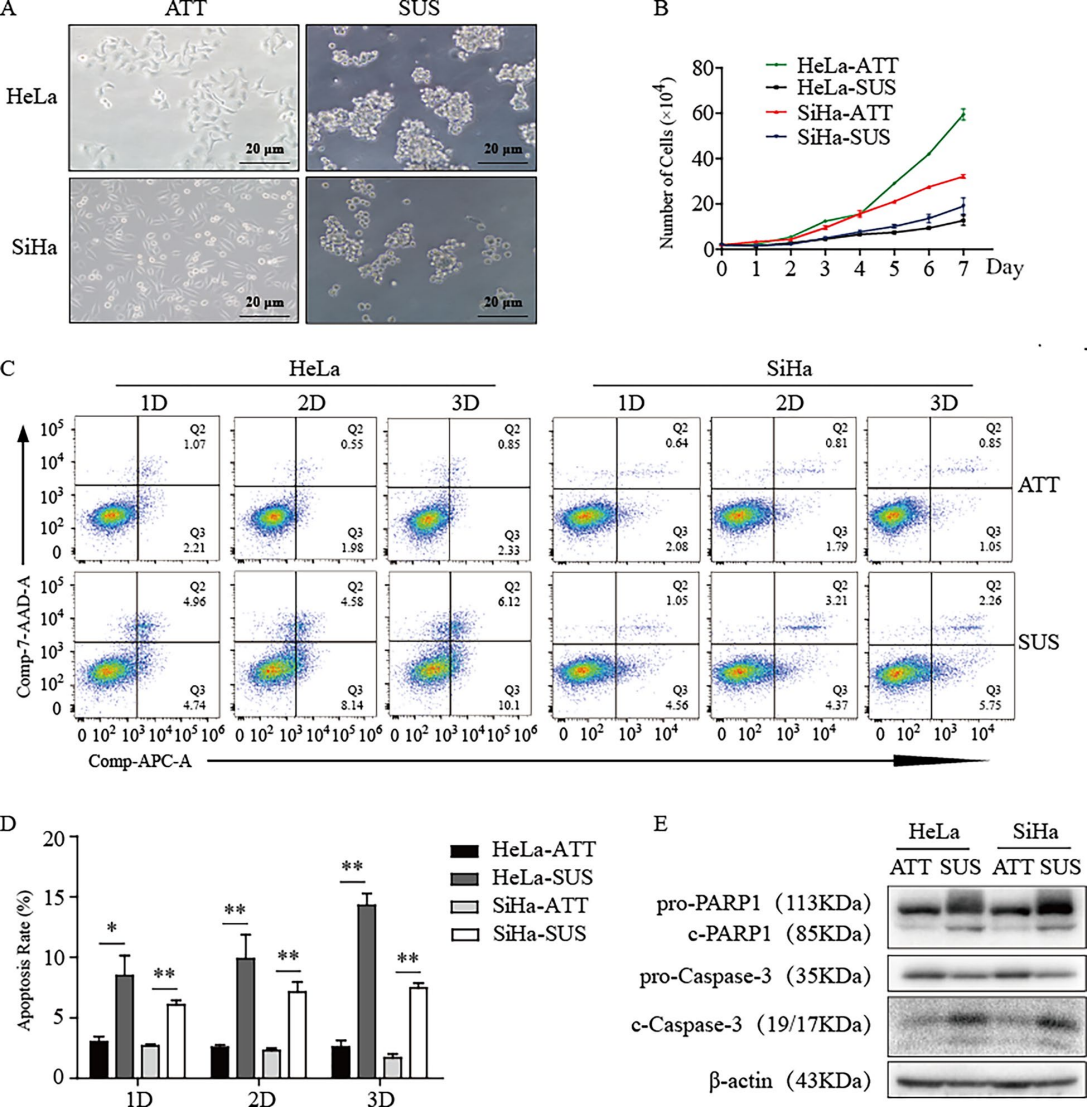
Constructing the model of anoikis.** A** Representative picture of the cell anoikis model.** B** Cell growth curve in different culture conditions by cell counting.** C** Flow cytometry assays of the apoptosis of HeLa and SiHa cells in ATT and SUS, respectively. **D** Statistical analysis of the percentage of the Q2 area added to the Q3 area in different culture conditions and different cells. **E** The expression level of apoptosis-associated proteins after 2 days in ATT or SUS. * P < 0.05, ** P < 0.01 (n = 3)

We know that the prerequisite of distant metastasis of tumor cells is the ability to overcome anoikis, which is a critical step. Therefore, we explored the relationship between Nrf2 and anoikis. First, we constructed a model of anoikis in HeLa and SiHa cells following the above method, and representative pictures are shown in Fig. 5A. We found that the morphology of cells changed obviously in suspension conditions (SUS) compared with attached conditions (ATT), which changed from long spindles with pseudopodia to oval shapes. The rates of cell growth were decreased significantly in SUS compared with ATT (Fig. 5B). We confirmed that this phenomenon was partly due to anoikis through flow cytometry assays, which showed that SUS greatly increased the number of apoptotic HeLa and SiHa cells after 2 days (Fig. 5C, D). Then, we verified our hypothesis by culturing cells in ATT or SUS for 2 days and detecting the expression of apoptosis-associated proteins such as PARP1 (Poly (ADP-Ribose) Polymerase-1) and Caspase-3 by western blot analysis. The results showed that the expression of pro-PARP1 and pro-Caspase-3 was decreased and that of c-PARP1 and c-Caspase-3 were increased significantly in SUS compared with ATT (Fig. 5E). These results showed that our anoikis model was successful.

### The level of Nrf2 impacted anoikis in HeLa and SiHa cells.

**Fig. 6:**
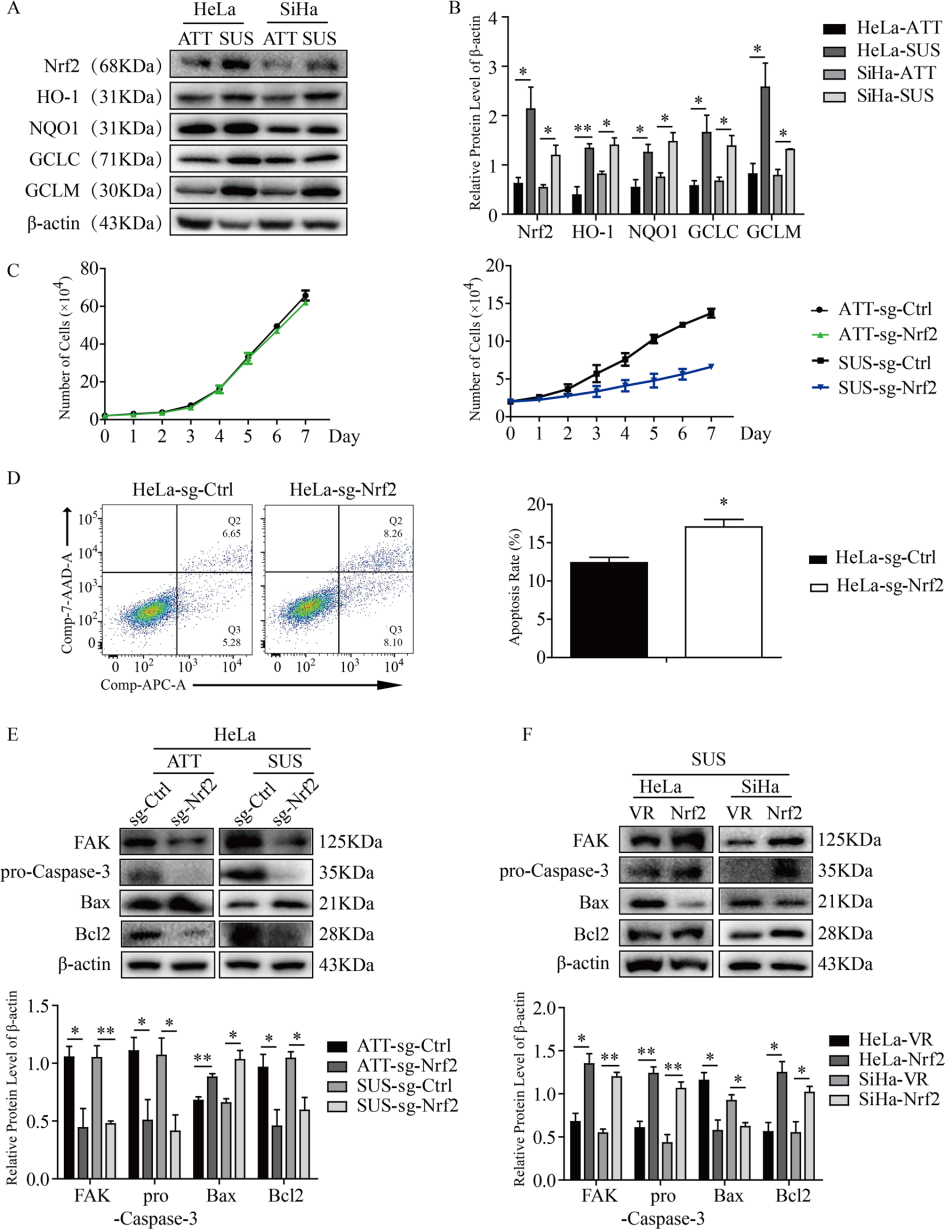
Nrf2 influenced anoikis in HeLa and SiHa cells. **A** The protein levels of Nrf2, HO-1, NQO1, GCLC and GCLM in HeLa and SiHa cells in ATT and SUS were measured. **B** Quantitative analysis of proteins. **C** The numbers of surviving cells in the HeLa sg-Ctrl group and HeLa sg-Nrf2 group were counted under different conditions. **D** The apoptotic percentage of HeLa sg-Ctrl and sg-Nrf2 cells in ATT and SUS through flow cytometry assays. **E** Testing the expression of Bax, Bcl2, FAK and pro-Caspase-3 in Nrf2 knockout HeLa cells and control cells in ATT and SUS by western blot. **F** Detection of the expression of Bax, Bcl2, FAK and pro-Caspase-3 in Nrf2-overexpressing cells in the SUS state. * P < 0.05, ** P < 0.01 (n = 3)

Next, we compared the protein expression of Nrf2 and its downstream molecules, including HO-1, NQO1, GCLC and GCLM, in ATT and SUS. The results showed that the protein levels of Nrf2 and its downstream molecules were increased significantly in SUS compared with ATT (Fig. 6A, B), which indicated that the Nrf2 signaling pathway was partly activated in SUS. To further determine the role of Nrf2 in providing resistance to anoikis in CC cells, we observed the proliferation of HeLa sg-Ctrl and HeLa sg-Nrf2 cells in ATT and SUS for 7 continuous days. Compared with HeLa sg-Ctrl cells, the proliferation of cells of HeLa sg-Nrf2 was inhibited significantly in SUS (Fig. 6C). However, we did not observe a significant difference in ATT. And the results of flow cytometry assays verified Nrf2 knockout increased the apoptotic rate of HeLa cells in SUS (Fig. 6D). Additionally, we measured the expression level of apoptosis-associated proteins in the above two different conditions. The results showed that, compared with ATT, Nrf2 knockout led to higher expression of Bax and lower expression of Bcl2, pro-Caspase-3 and FAK, which are important anoikis resistant molecules in SUS (Fig. 6E). In contrast, overexpression of Nrf2 resulted in higher FAK, Bcl2 and pro-Caspase-3 but lower Bax in SUS (Fig. 6F). All of the above results suggested that Nrf2 enhanced the resistance of HeLa and SiHa cells to anoikis but not general apoptosis.

### Nrf2 knockout inhibited lung metastasis and lymph node metastasis in vivo.

**Fig. 7:**
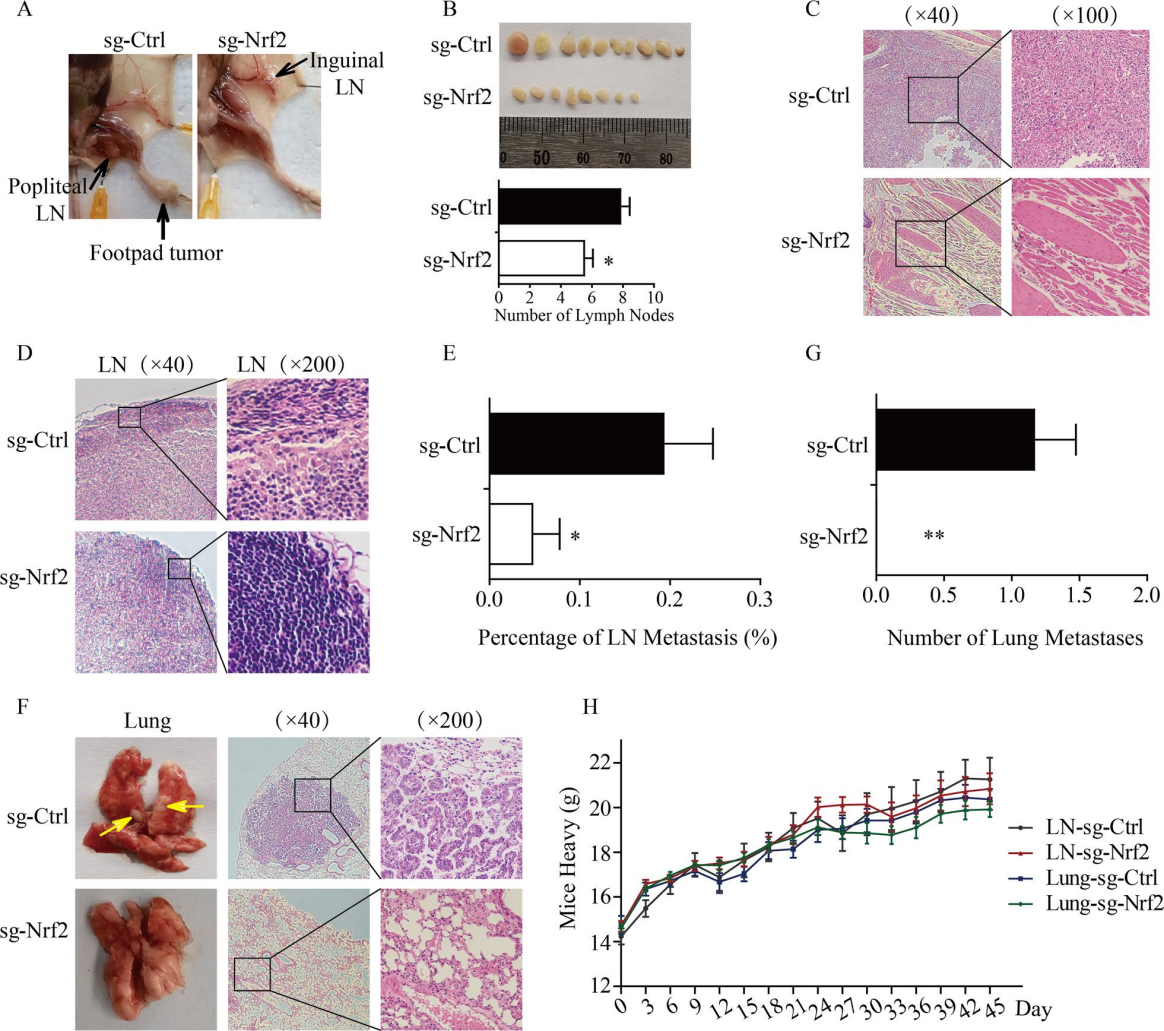
Nrf2 facilitated the metastasis of HeLa cervical cancer cells in vivo. **A** Representative picture of the lymph node metastasis model. **B** The number of macroscopic lymph nodes in the HeLa sg-Ctrl group and sg-Nrf2 group. **C** Representative pictures of footpad tumors through HE staining. **D** Representative images of HE staining of lymph nodes. **E** The rate of lymph node metastasis. **F** Representative photos of HE staining of lungs. **G** The number of lung metastases. **H** The weight of mice in different groups. * P < 0.05, ** P < 0.01 (n = 6)

To further investigate the effects of Nrf2 on metastasis in vivo, xenograft mouse metastatic models were established. In the xenograft mouse lymph node metastasis model, we found that the volumes of popliteal lymph nodes and inguinal lymph nodes were smaller and the number of macroscopic lymph nodes was lower in the Nrf2-knockout group than in the control group (Fig. 7A, B). Figure 7C shows representative HE pictures of footpad tumors in the control group. In addition, the incidence of lymph node metastasis dramatically declined in the Nrf2 knockout group (Fig. 7D, E). Moreover, we observed macroscopic and HE metastases in the control group; however, the Nrf2-knockout group had no marked metastases in the xenograft mouse lung metastasis model (Fig. 7F, G). About body weight of mice, there was no statistically significant difference among different groups (Fig. 7H). These results confirmed that knockout of Nrf2 could obviously inhibit the distant metastasis of HeLa cells in vivo.

### Nrf2 enhanced resistance to anoikis in cervical cancer cells by promoting the expression of Snail1.

**Fig. 8:**
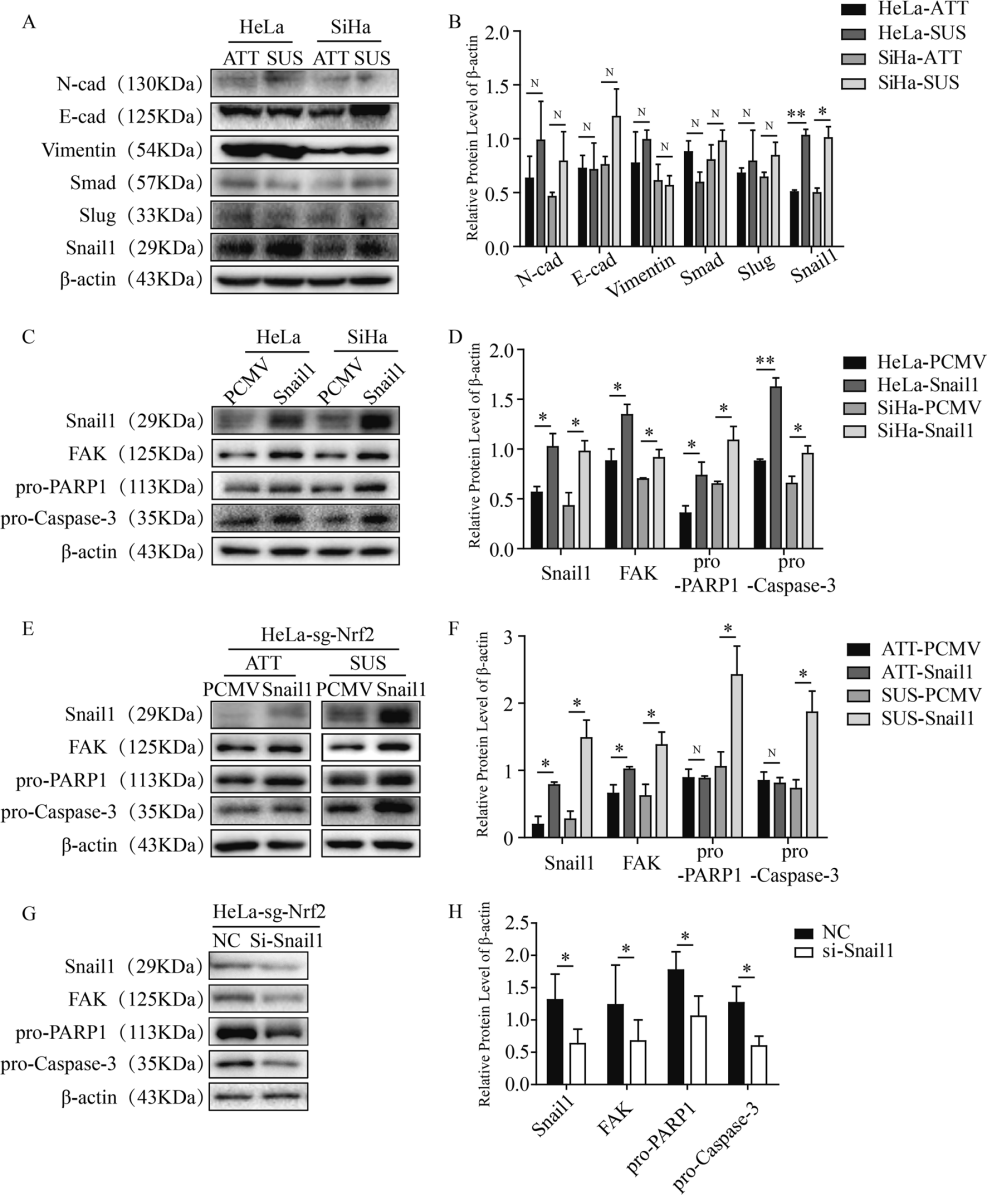
Snail1 plays an important role in the resistance to anoikis caused by Nrf2 in cervical cancer cells. **A** Measuring the levels of EMT-associated proteins, including N-cad, E-cad, Snail1, Slug, Smad and Vimentin, under ATT and SUS conditions. **C** The expression of FAK, pro-Caspase-3 and pro-PARP1 when Snail1 was overexpressed in the SUS state. **E** Overexpressing Snail1 in Nrf2 knockout HeLa cells and testing the levels of FAK, pro-Caspase-3 and pro-PARP1 under different conditions. **G** Snail1 siRNA was used to treat HeLa sg-Nrf2 cells, and the expression of FAK, pro-Caspase-3 and pro-PARP1 was measured. **B**, **D, F**, **H** The protein expression levels were quantitatively analyzed. * P < 0.05, ** P < 0.01 (n = 3)

Although the above results indicated that the higher level of Nrf2 could enhance the EMT process and resistance to anoikis in HeLa and SiHa cells, the specific mechanisms have not been clarified, and the relationship between EMT and anoikis under different Nrf2 expression levels has not been elucidated. We found that the expression of Nrf2 was increased under SUS conditions (Fig. 6A), and then, we observed the expression of EMT-associated proteins in the SUS state. Interestingly, compared with ATT, the expression of Snail1 was increased significantly under SUS conditions, while other proteins, including E-cad, N-cad, Slug, Smad, and Vimentin, were not consistently changed (Fig. 8A, B). Previous studies have verified that Snail1 is the key regulator of the EMT process. Therefore, we hypothesized that Nrf2 promoted the resistance of HeLa and SiHa cells to anoikis-mediated cell death by promoting the expression of Snail1. To confirm this hypothesis, we momentarily overexpressed Snail1 in HeLa and SiHa cells through transfected Snail1 plasmid and cultured the cells under SUS conditions. We found that the level of FAK, pro-Caspase-3 and pro-PARP1 was increased significantly when Snail1 was overexpressed (Fig. 8C, D). To further prove the role of Snail1 in resistance to anoikis, which was enhanced by Nrf2, we overexpressed Snail1 in Nrf2 knockout HeLa cells. Surprisingly, the lower FAK, pro-Caspase-3 and pro-PARP1 levels attributed to Nrf2 knockout were partly reversed under SUS conditions (Fig. 8E, F). We used Snail1 siRNA to treat HeLa sg-Nrf2 cells, and the results showed that the expression levels of FAK, pro-Caspase-3 and pro-PARP1 were further decreased (Fig. 8G, H). In summary, Nrf2 enhances resistance to anoikis in cervical cancer cells by promoting the expression of Snail1.

## Discussion

Approximately 46.4% of cervical cancer patients have a five-year survival rate of 91.5% after standard treatment. Unfortunately, metastasis of cervical cancer cells causes this rate to drop to 57.4% [30]. Therefore, uncovering the molecular mechanisms underlying metastasis may provide therapeutic strategies for cervical cancer patients with metastasis.

A series of studies have proved that oxidative stress and the imbalance between ROS and antioxidant defense mechanisms were implicated in the initiation, development and metastasis of cancer [31]. Oxidative stress can activate many transcription factors, and Nrf2 is arguably the most important regulator of the expression of molecules that have antioxidant functions within the cell [32, 33]. Subsequently, as a transcription factor and regulator of many antioxidant genes and antioxidant enzymes, Nrf2 protects cells from oxidative or electrophilic stress by activating downstream target genes and enzymes such as HO-1, NAD(P)H quinone oxidoreductase 1 (NQO1), glutamate-cysteine ligase catalytic subunit (GCLC), glutamate-cysteine ligase modifier subunit (GCLM), catalase (CAT), superoxide dismutase (SOD), and glutathione peroxidase (GPx) [34]. Conversely, in the development of cancer, Nrf2 plays the facilitating role. And in our work, Nrf2 and its downstream molecules, including GCLC, GCLM, NOQ1 and HO-1 were increased in the suspension and avoiding anoikis, although we did not measure the levels of SOD, CAT or GPx, simultaneously. A previous study showed that Nrf2 was overexpressed in cervical cancer compared with the normal cervix and was associated with an unsatisfactory prognosis [25]. However, the eyes primarily focused on the interaction of Kelch-like ECH-associated protein 1 (Keap1) and Nrf2 [35, 36]. Herein, we investigated the important role of crosstalk among Nrf2, EMT and anoikis in the metastasis of cervical cancer. Our preliminary experiments demonstrated that Nrf2 was more highly upregulated in cervical cancer patients with lymph node metastasis. Moreover, the migration ability of cervical cancer HeLa and SiHa cells was positively correlated with the level of Nrf2 in vitro. Certainly, the xenograft tumor model of Nrf2 knockout in HeLa cells also supported the above views.

In addition to genetics and external environmental factors, the transformation under mechanical forces and the physical interaction of cancer cells with their microenvironment are key determinants of cancer cell metastasis [37]. Epithelial-mesenchymal transition has been widely studied because of its involvement in the malignant behaviors of cancer cells in the tumor microenvironment. During EMT, cancer cells lose cell–cell contact and gain mesenchymal properties to increase invasiveness. It has been verified that the loss of E-cadherin, an intercellular adhesion molecule, resulted in dramatic changes in the physical and morphological properties of cancer cells, which exhibited a reduction in intercellular adhesion and a change from cubic to spindle epithelium of cell morphology [38]. As a mesenchymal marker, Vimentin can regulate the interaction of cytoskeleton proteins and cell adhesion molecules to promote invasion and repress metastatic outgrowth [39, 40]. Consistently, our study showed that the knockout of Nrf2 significantly increased the expression level of E-cad and decreased that of Vimentin, which inhibited metastasis in lymph nodes and lungs in vivo. These data revealed that the level of Nrf2 was positively associated with EMT in cervical cancer. Moreover, the expression of other mesenchymal factors, such as N-cadherin (N-cad) [41], Snail1 (Snail) [42] and Snail2 (Slug) [43], was also decreased in the absence of Nrf2. Therefore, we hypothesized that Nrf2 could promote the metastasis of cervical cancer cells by promoting the process of EMT.

In addition, our finding of the effect of Nrf2 on anoikis in cervical cancer is consistent with previous studies that suggested Nrf2 and its downstream molecules promote the metastasis of cancer cells by enhancing anoikis resistance [22, 24, 44, 45]. We verified the success of the anoikis model of cervical cancer cells through changes in the precursors and shears of PARP1 and Caspase-3 since PARP1 is the marker of apoptosis and the primary target of Caspase-3 [46]. The expression of Nrf2 and its downstream molecules was increased in suspension, which supported that the signaling pathway of Nrf2 was activated in suspension and was consistent with oxidative stress. However, knockout of Nrf2 significantly increased the death of HeLa cells in suspension rather than in attached conditions. These results indicated that Nrf2 could promote resistance to anoikis in cervical cancer. In addition, Snail1 has been widely concerned because of its transcriptional regulatory capacity in EMT and its function of invasion in cancer cells [47]. Interestingly, our further results showed that the expression of Snail1 was significantly increased compared with that of other EMT-associated molecules in suspension, and the level of Snail1 could influence anoikis in cervical cancer cells. Rescued experiments showed that overexpressing Snail1 could increase the levels of FAK, pro-PARP1 and pro-Caspase-3, which were decreased by Nrf2 knockout in HeLa cells. Therefore, we confirmed that Nrf2 increased resistance to anoikis by promoting the expression of Snail1. However, Ma et al. hypothesized that the inactivation of FAK could reduce the occurrence of EMT by increasing E-cad and reducing Snail1 [48]. Perhaps there is a bidirectional ring between FAK and Snail1 in EMT and anoikis, which needs further research.

## Conclusion

In this research, we revealed that Nrf2 enhanced EMT and anoikis resistance by promoting the expression of Snail1 and finally accelerated the metastasis of HeLa and SiHa cervical cancer cells. Inhibiting the expression of Nrf2 may be a novel target to inhibit metastasis in cervical cancer.

## Data Availability

Data related to the current study are available from the corresponding author on reasonable request.

## Abbreviations

CC: Cervical cancer
EMT: Epithelial-mesenchymal transition
E-cad: E-cadherin
N-cad: N-cadherin
FAK: Focal adhesion kinase
Nrf2: Nuclear factor erythroid 2-related factor 2
ROS: Reactive oxygen species
IHC: Immunohistochemistry
HO-1: Heme oxygenase 1
GCLC: Glutamate-cysteine ligase catalytic subunit
GCLM: Glutamate-cysteine ligase modifier subunit
NQO1: NAD(P)H quinone oxidoreductase 1
PARP1: Poly (ADP-Ribose) Polymerase-1
H&E: Hematoxylin–eosin
SUS: Suspension conditions
ATT: Attached conditions
CAT: Catalase
SOD: Superoxide dismutase
GPx: Glutathione peroxidase
Keap1: Kelch-like ECH-associated protein 1
