# Correction: the promoting effect and mechanism of Nrf2 on cell metastasis in cervical cancer

**DOI:** 10.1186/s12967-025-06953-x

**Published:** 2025-12-03

**Authors:** Mengwen Zhang, Xiaoling Hong, Ning Ma, Zhentong Wei, Xinxin Ci, Songling Zhang

**Affiliations:** 1 Department of Obstetrics and Gynecology, The First Hospital of Jilin University, Changchun, China; 2 Institute of Translational Medicine, The First Hospital of Jilin University, Changchun, China

**Correction to : J Transl Med 21, 433 (2023).** 10.1186/s12967-023-04287-0

Following publication of the original article [1], the authors reported that Fig. 2A lower left image was inadvertently reused in Fig. 5A lower left image.

The incorrect version of Fig. 5 was:

**Fig. 5:**
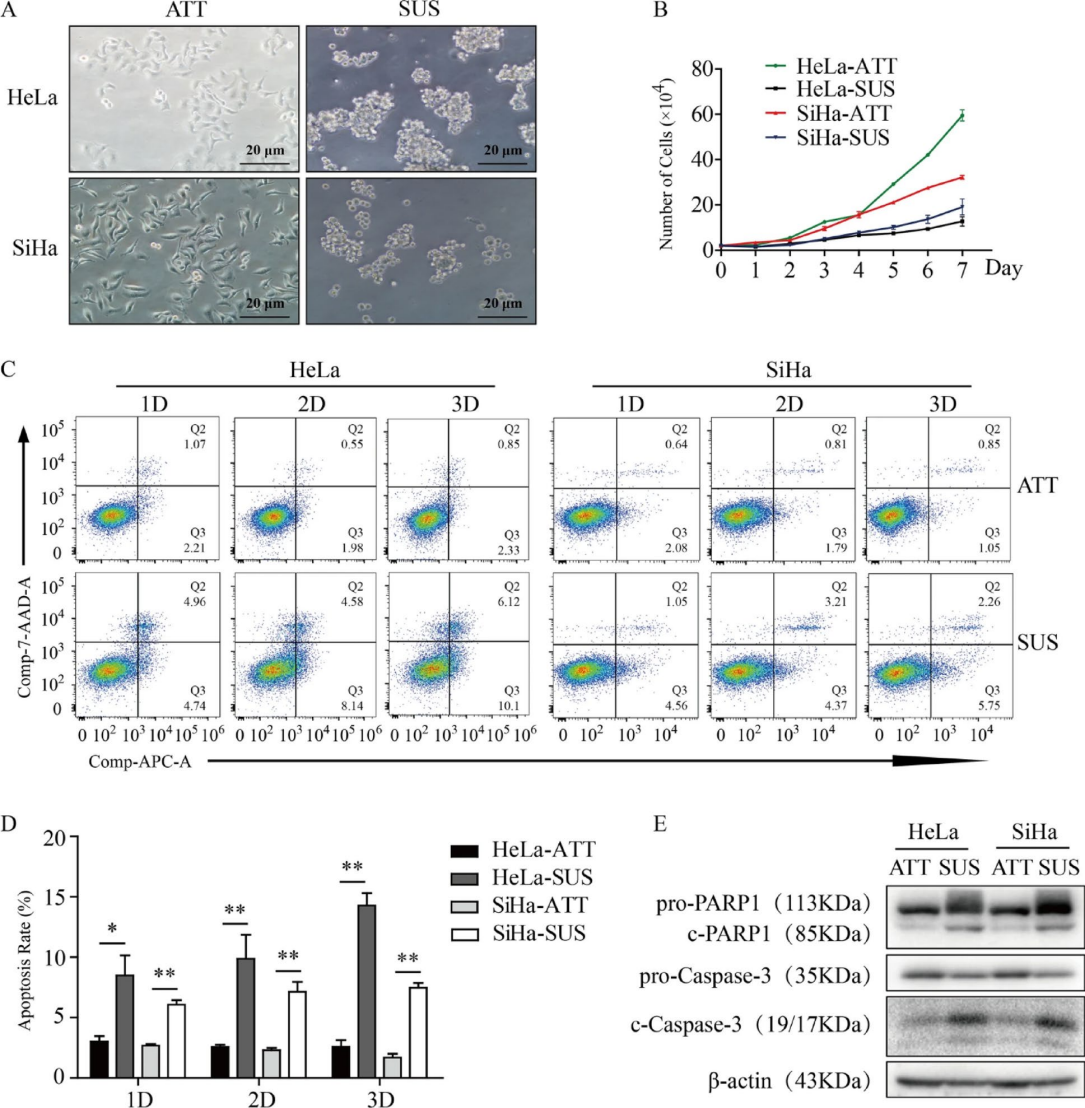
Constructing the model of anoikis. **A** Representative picture of the cell anoikis model. **B** Cell growth curve in different culture conditions by cell counting. **C** Flow cytometry assays of the apoptosis of HeLa and SiHa cells in ATT and SUS, respectively. **D** Statistical analysis of the percentage of the Q2 area added to the Q3 area in different culture conditions and different cells. **E** The expression level of apoptosis-associated proteins after 2 days in ATT or SUS. * *P* < 0.05, ** *P* < 0.01 (*n* = 3)

The correct Fig. 5 is:

**Fig. 5:**
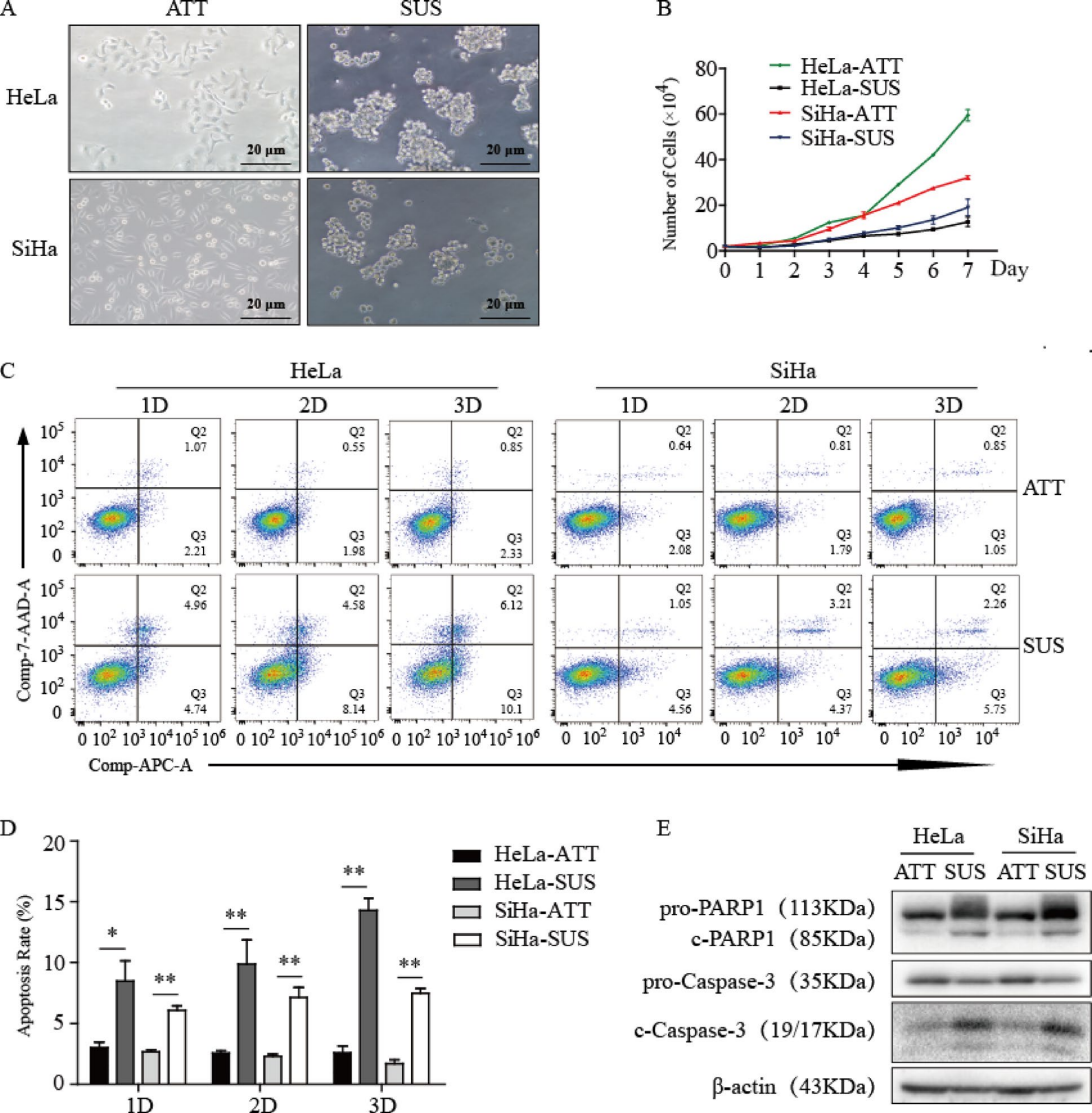
Constructing the model of anoikis. **A** Representative picture of the cell anoikis model. **B** Cell growth curve in different culture conditions by cell counting. **C** Flow cytometry assays of the apoptosis of HeLa and SiHa cells in ATT and SUS, respectively. **D** Statistical analysis of the percentage of the Q2 area added to the Q3 area in different culture conditions and different cells. **E** The expression level of apoptosis-associated proteins after 2 days in ATT or SUS. * *P* < 0.05, ** *P* < 0.01 (*n* = 3)

The original article [1] has been updated.
